# Adsorption Characteristics of Dimethylated Arsenicals on Iron Oxide–Modified Rice Husk Biochar

**DOI:** 10.3390/toxics10110703

**Published:** 2022-11-17

**Authors:** Sang-Gyu Yoon, Ihn-Sil Kwak, Hye-On Yoon, Jinsung An

**Affiliations:** 1 Department of Environment Safety System Engineering, Semyung University, Jecheon 27136, Republic of Korea; 2 Department of Ocean Integrated Science, Chonnam National University, Yeosu 59626, Republic of Korea; 3 Korea Basic Science Institute, 145 Anam-ro, Seoul 02841, Republic of Korea; 4 Department of Civil & Environmental Engineering, Hanyang University, Ansan 15588, Republic of Korea

**Keywords:** dimethylmonothioarsinic acid, dimethyldithioarsinic acid, adsorption isotherm, X-ray absorption spectroscopy, linear combination fitting

## Abstract

In this study, the adsorption characteristics of dimethylated arsenicals to rice husk biochar (BC) and Fe/biochar composite (FeBC) were assessed through isothermal adsorption experiments and X-ray absorption spectroscopy analysis. The maximal adsorption capacities (q_m_) of inorganic arsenate, calculated using the Langmuir isotherm equation, were 1.28 and 6.32 mg/g for BC and FeBC, respectively. Moreover, dimethylated arsenicals did not adsorb to BC at all, and in the case of FeBC, q_m_ values of dimethylarsinic acid (DMA(V)), dimethylmonothioarsinic acid (DMMTA(V)), and dimethyldithioarsinic acid (DMDTA(V)) were calculated to be 7.08, 0.43, and 0.28 mg/g, respectively. This was due to the formation of iron oxide (i.e., two-line ferrihydrite) on the surface of BC. Linear combination fitting using As K-edge X-ray absorption near edge structure spectra confirmed that all chemical forms of dimethylated arsenicals adsorbed on the two-line ferrihydrite were DMA(V). Thus, FeBC could retain highly mobile and toxic arsenicals such as DMMTA(V) and DMDTA(V)) in the environment, and transform them into DMA(V) with relatively low toxicity.

## 1. Introduction

Arsenic (As) is found in the environment (water, soil, and air) as a result of both natural and anthropogenic factors, and is considered a health risk factor worldwide due to its high toxicity and carcinogenicity not only to animals but also to humans [1,2,3,4]. The toxicity and mobility of As in the environment are greatly affected by the species [5,6,7,8], which can generally be classified into inorganic arsenicals (e.g., arsenate [As(V)] and arsenite [As(III)]) and organoarsenicals (e.g., dimethylarsinic acid [DMA(V)], dimethylmonothioarsinic acid [DMMTA(V)], and dimethyldithioarsinic acid [DMDTA(V)]). Organoarsenicals can be formed via the methylation of inorganic arsenicals, a process known as the detoxification [9]. Although the toxicities of inorganic arsenicals are greater than those of organoarsenicals [2,10], interest in dimethylated arsenicals (particularly DMMTA(V) and DMDTA(V)) has grown; this is because recent studies have demonstrated that the half maximal inhibitory concentrations (IC50) of As(V), DMA(V), and DMMTA(V) against human epidermoid carcinoma A431 cells were 571, 843, and 10.7 μM, respectively [11]. Another study reported that the 48 h acute toxicities (IC50) of As(V), DMA(V), DMMTA(V), and DMDTA(V) against *Daphnia magna* were 9.5, <30, 1.7, and 6.5 mg/L, respectively [12]. Dimethylated arsenicals are potential human and ecological risks, as they have been detected in various environments such as geothermal waters, paddy pore waters, landfill leachate, and peatlands [13,14,15,16,17], as well as in foods such as seafood, rice grains, and other rice products [18,19,20].

Biochar is produced via pyrolysis of various biomass, such as agricultural waste and sewage sludge. It has the ability to remove heavy metals and organic contaminants from soil and water due to its high surface area and abundant surface functional groups [4,21,22,23,24,25,26,27]. However, oxygen-containing functional groups, such as carboxylic (-COOH), carbonyl (-COH), and hydroxyl (-OH) groups, render the surface charge of biochar negative [28,29,30,31]. As a result, electrostatic repulsion can negatively affect the adsorption of anionic contaminants. To overcome this drawback, several previous studies have used biochar modified with iron (Fe) oxides, and subsequently observed significant increases in the adsorption capacity for anionic contaminants [32,33,34,35,36,37,38,39]. Fe oxides exist ubiquitously throughout the environment and also exhibit a high binding affinity for anionic contaminants such as As [40,41,42,43,44,45,46,47]. Kim et al. [48] assessed the adsorption capacity of As(III) by modifying *Miscanthus* biochar with Fe. The results of their study showed that the adsorption proportion of As(III) for unmodified biochar and biochar modified with Fe were <10.4% and >72.3%, respectively, confirming that the adsorption capacity increased approximately 7-fold after modification with Fe. Furthermore, Wang et al. [32] analyzed the As(V) adsorption capacity of *Pinus taeda* biochar modified with hematite, which was increased by approximately 1.6-fold after Fe modification. The maximum As(V) adsorption of biochar before and after modification was 265.2 and 428.7 mg/kg, respectively, confirming that the As(V) adsorption capacity after Fe modification increased by about 1.6 folds. In addition, a notable disadvantage of unmodified biochar was that it was difficult to separate and reuse [49]; whereas when modified with Fe oxides, biochar could be separated using magnetization (i.e., ease of recovery). Phosphate recovery efficiency was recorded as 70.4% even after five cycles of reuse, indicating that it could be used as a more efficient adsorbent compared to other phosphate adsorbent materials [50]. Consequently, interest in biochar modified with such Fe oxides has continued to increase [51,52].

Here, we aimed to assess the effectiveness of rice husk biochar (BC) as a tool for reducing the mobility and toxicity of dimethylated arsenicals in the environment. In particular, the change in adsorption capacity was observed by modifying BC with two-line ferrihydrite, and the chemical forms of adsorbed dimethylated arsenicals were subsequently assessed by using X-ray absorption spectroscopy (XAS). As there have been few studies on the adsorption of dimethylated arsenicals from using Fe oxides and BC compared to those on inorganic arsenicals, this study aimed to improve the understanding of the adsorption characteristics of Fe/biochar composite (FeBC) for dimethylated arsenicals. Furthermore, the findings of this study can provide justification for the inclusion of the use of FeBC as part of risk mitigation measures for water and/or soil contaminated with dimethylated arsenicals.

## 2. Materials and Methods

### 2.1. BC

The BC used in this study was prepared by thermally decomposing rice husk at 500 °C and was provided by the Water Quality Laboratory of Seoul National University (South Korea). The physicochemical characteristics of BC were analyzed in a previous study [53], which revealed a mean pore size of 221 nm, a specific surface area of 24.34 m^2^/g, an intra-particle porosity of 41.71%, and a skeletal density of 1.69 g/cm^3^. The respective concentrations of C and O were 47.82% and 8.98%, whilst the O/C ratio was 0.14. The provided BC was finely ground by using a pestle and subsequently stored in a desiccator. The particle size of the ground BC was analyzed by using a particle-size analyzer (Partica mini LA 350, Horiba, Japan).

### 2.2. Synthesis of FeBC

The synthesis of FeBC was performed according to a partially modified two-line ferrihydrite synthesis method that has been previously reported [54]. Briefly, 15 g of BC was added to 150 mL of 0.1 M iron(III) nitrate nonahydrate solution (Fe(NO_3_)_3_·9H_2_O, ≥99%, Daejung, Siheung-si, Republic of Korea); 1 M NaOH was then added to adjust the pH to 7.5. Distilled water was added to increase the volume of the solution to 200 mL. This prepared solution was then reacted for 24 h at room temperature, with intermittent stirring, and then solid–liquid separation was performed by using a vacuum filter (JS-2D, JEIL, Seoul, Republic of Korea). The recovered solid was washed several times using distilled water, dried at room temperature, and then stored in a desiccator. Elemental mapping was performed by using field-emission scanning electron microscopy (FE-SEM) equipped with an energy-dispersive X-ray spectrometer (EDS) (SU8220, Hitachi, Tokyo, Japan) to analyze Fe on the surface of the synthesized FeBC. The particle size of FeBC was analyzed by using the particle size analyzer, and the total Fe concentration of FeBC was measured via digestion with aqua regia. FeBC (1 g) was placed in a 15 mL polypropylene tube, and 10 mL of aqua regia (7.5 mL of HCl and 2.5 mL of HNO_3_) was added, followed by reaction at room temperature for 24 h, and finally filtering through a 0.45 μm GHP filter (Pall Corporation, Port Washington, NY, USA). Fe content analysis in this filtered solution was performed at 248.3 nm, using an atomic absorption spectrometer (AAS) (AAS-6200, Simadzu, Japan).

### 2.3. Equilibrium Adsorption Isotherm Experiments

Initial concentrations were prepared as follows; 10, 100, 200, 500, 1000, and 2000 mg/L for As(V) (Na_2_HAsO_4_·7H_2_O, ≥99%, Wako, Japan); 10, 100, 200, 500, 1000, 2000, and 5000 mg/L for DMA(V) (C_2_H_7_AsO_7_, ≥99%, Sigma-Aldrich, St. Louis, MO, USA); 0.5, 5, 10, 20, 50, and 100 mg/L for DMMTA(V) (C_2_H_7_AsOS, 95%, Toronto Research Chemicals, Toronto, Canada); and 0.5, 1, 5, 10, 20, and 50 mg/L for DMDTA(V) (C_2_H_7_AsNaS_2_;, 97%, Toronto Research Chemicals, Toronto, Canada). Adsorption isotherm experiments were conducted after adjusting the pH to 7.0, using 0.1 M HCl and 1 M NaOH. A solution containing 0.1 g of BC or FeBC and each As species (10 mL) was placed in a 50 mL polypropylene tube, stirred at 120 rpm for 24 h, and then filtered by using a 0.45-μm GHP filter. Thereafter, the concentration of As in the solution was analyzed at 196.696 nm, using an inductively coupled plasma–optical emission spectrometer (ICP–OES) (Ultima 2C, Horiba Jobin Yvon, Vénissieux, France).

To obtain the parameters that could explain adsorption characteristics, including maximum adsorption capacity (q_m_), for each As species, three different adsorption isotherm models (i.e., Langmuir isotherm, Freundlich isotherm, and Temkin isotherm) were applied here. In our preliminary results (Supplementary Table S1), the Langmuir isotherm (Equation (1)) showed the highest R^2^ values for dimethylated arsenicals, and therefore, its parameters were derived and used for the assessment of adsorption characteristics here.

(1) $$q_{e}=\frac{q_{m}{\mathrm{bC}}_{e}}{1+{\mathrm{bC}}_{e}}$$

where q_e_ is the concentration of As adsorbed to the solid at equilibrium (mg/g), b is the Langmuir constant (L/mg), and C_e_ is the concentration of As remaining in the liquid at equilibrium (mg/L).

### 2.4. XAS

As K-edge XAS data were collected at the 10C wide XAFS beamline in the Pohang Accelerator Laboratory, Korea, in fluorescence mode. To investigate the adsorption characteristics of each As species (As(V), DMA(V), DMMTA(V), and DMDTA(V)) on FeBC (i.e., to confirm the chemical form of each As species adsorbed onto the two-line ferrihydrite formed on the BC surface), raw spectra data were pretreated by using the Athena software 0.2.96, which involved the normalization of edge-step and background removal. This was followed by linear combination fitting (LCF), using four kinds of As reference compounds (As(V), DMA(V), DMMTA(V), and DMDTA(V)). In addition, the suitability of LCF fitting was evaluated by using the R-factor value [55,56].

## 3. Results and Discussion

### 3.1. Characteristics of FeBC

**Figure 1:**
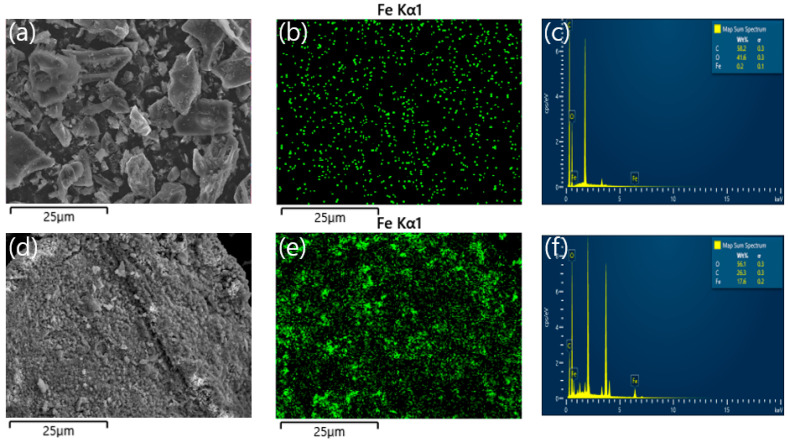
FE-SEM images and EDS elemental mapping results of BC and FeBC: (**a**) FE-SEM image of BC, (**b**,**c**) elemental mapping results of BC, (**d**) Fe-SEM image of FeBC, and (**e**,**f**) elemental mapping results of FeBC.

Figure 1 shows the FE-SEM images and elemental mapping results for the surfaces of BC and synthesized FeBC. The Fe compositions of BC and FeBC surfaces, detected via elemental mapping, were measured to be 0.2% (wt.%) and 17.6% (wt.%), respectively (Figure 1c,f and Table 1). In addition, the result of aqua regia digestion showed that the total Fe concentrations in BC and FeBC were 527 and 47,155 mg/kg, respectively (Table 1). The difference in the Fe composition of BC and FeBC surfaces confirmed via elemental mapping (88 folds; 0.2% and 17.6%, respectively) and that confirmed via aqua regia digestion (89.5 folds; 527 and 47,155 mg/kg, respectively) were similar, thus suggesting that Fe oxides’ precipitates were well dispersed across the FeBC surface (Figure 1b,e). The median particle size of FeBC was approximately 56 μm.

### 3.2. Comparison of Adsorption Characteristics of Arsenicals on BC and FeBC

**Figure 2:**
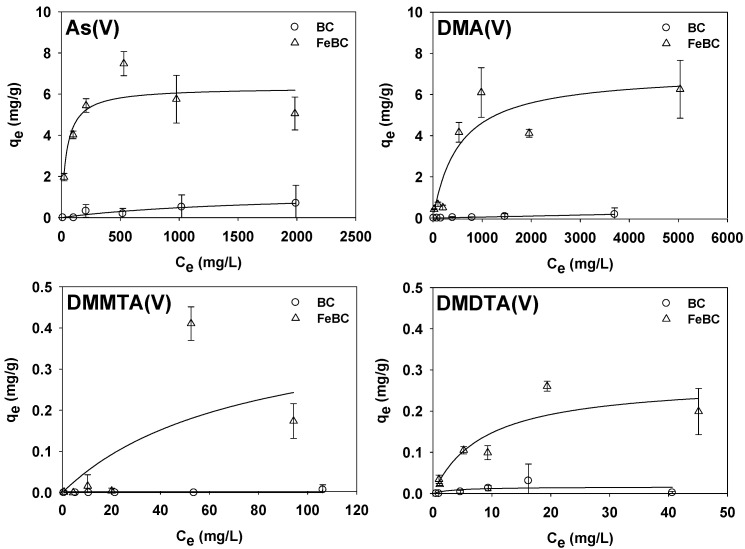
Results of adsorption isotherm experiments for each As species (As(V), DMA(V), DMMTA(V), and DMDTA(V)) for BC and FeBC. The maximal adsorption capacity (q_m_ [mg/g]) and coefficient of determination (R^2^) of As(V) for BC were 1.28 and 0.2821, respectively. DMA(V), DMMTA(V), and DMDTA(V) were not adsorbed onto BC. For FeBC, the q_m_ (mg/g) values of As(V), DMA(V), DMMTA(V), and DMDTA(V) were 6.32, 7.08, 0.43, and 0.28, respectively, whilst R^2^ values were 0.6837, 0.7920, 0.5075, and 0.7724, respectively.

The q_m_ of As(V), which was obtained via Langmuir isotherm fitting, was calculated as 1.28 mg/g on BC and 6.32 mg/g on FeBC (Figure 2). The q_m_ of As(V) on FeBC was significantly increased compared to that on BC, which indicated that Fe oxides formed on the surface of BC could serve as adsorption sites with a high efficiency for As(V) [32]. Most BC surfaces are negatively charged due to the presence of oxygen-containing functional groups such as -COO; therefore, it is difficult for them to adsorb anionic contaminants such as As due to electrostatic repulsion [35,36,57,58,59]. However, BC modified with Fe can increase the adsorption capacity for anionic contaminants, such as As, by neutralizing the surface charge of BC, and thereby providing a positive adsorption site on the BC surface [33]. Feng et al. [33] conducted adsorption isotherm experiments for reactive red 120 (RR120), an anionic contaminant, at pH 3.5 and 7.5, using the Hickory chip (HC) and Fe-loaded Hickory chip (FeHC). As a result, it was confirmed that the q_m_ of RR120 for FeHC increased to 32.0 mg/g at pH 3.5 (2.90 mg/g for HC) and to 79.4 mg/g at pH 7.5 (2.39 mg/g for HC). It was concluded that the increase in the surface charge of HC after Fe loading (i.e., the point of zero charges (pzc) increased from about 2 to 9.19) accelerated the electrostatic interaction between RR120 and the adsorbent, thereby increasing the adsorption capacity. Additionally, Fan et al. [34] performed adsorption isotherm experiments on As(V) at pH 3.0, using corn straw (CS) and Fe-infused CS (CS-Fe). They subsequently found that the q_m_ of As(V) for CS-Fe increased to 14.77 mg/g, compared to 2.86 mg/g for CS, which was considered to be a result of the increase in adsorbent surface charge due to the increase in the pzc (from 2.75 to 6.93). He et al. [60] also performed adsorption isotherm experiments on As(V), using corn straw biochar (CSB) and Fe-infused CSB (FeCSB), and reported that the q_m_ values of As(V) for CSB and FeCSB at pH 7.0 ± 0.2 were 0.017 and 6.80 mg/g, respectively, suggesting an estimated 400-fold improvement resulting from an increase in pzc from 4.5 to 7.4. Table 2 summarizes the change in the anionic contaminant adsorption capacity before and after dropping Fe into BC.

It was expected that the pzc values of BC prepared by pyrolysis at 500 °C ranged from 5.6 to 7.0 [61,62]. In addition, as the pzc of general two-line ferrihydrite was 8.0 to 8.4 [63], the formation of two-line ferrihydrite on the BC surface caused a change in the electrostatic interaction, with an increase in the pzc of BC simultaneously, which in turn increased the adsorption capacity for As(V). Through processes such as post-synthetic modification, it is possible to produce materials (i.e., adsorbents) that have the characteristics of both organic and inorganic materials and compensate for each other’s disadvantages [64]. Adsorption capacity can be improved not only by electrostatic interaction but also by van der Waals interaction [65,66].

BC with a high porosity, high specific surface area, and abundant surface functional groups has been widely used to adsorb organic pollutants [67,68]. However, our observations revealed that dimethylated arsenicals (DMA(V), DMMTA(V), and DMDTA(V)) were not adsorbed to BC (Figure 2). We presumed this was caused by the aforementioned electrostatic repulsion (DMA(V), pK_a_ = 6.2; DMMTA(V), pK_a_ = 4.3, 6–7; and DMDTA(V), pK_a_ = 2.25) [69], as well as the effect of steric hindrance; however, further in-depth studies are required to clarify these aspects. In contrast, the q_m_ values of DMA(V), DMMTA(V), and DMDTA(V) to FeBC were calculated as 7.08, 0.43, and 0.28 mg/g, respectively (Figure 2). Consequently, Fe oxides (two-line ferrihydrite) formed on the FeBC surface were presumed to have been the main factor in the adsorption of DMA(V), DMMTA(V), and DMDTA(V). According to the results of adsorption isotherm experiments presented by Yoon et al. [12], using As(V), DMA(V), DMMTA(V), and DMDTA(V) with amorphous Fe oxide two-line ferrihydrite at different pH conditions, the q_m_ values of As(V), DMA(V), DMMTA(V), and DMDTA(V) at pH 7.0 were 61.6, 43.6, 14.0, and 2.5 mg/g, respectively. This indicated that the q_m_ of each As species to two-line ferrihydrite decreased as the methyl and thiol group content increased (DMMTA(V) has one thiol group, and DMDTA(V) has two thiol groups). Therefore, adsorption capacity was inhibited due to the presence of methyl and thiol groups. Similarly, the present study confirmed that the q_m_ for FeBC decreased as the thiol content in organoarsenic species increased.

Yoon et al. [12] studied the species transformation potential of DMA(V), DMMTA(V), and DMDTA(V) over 24 h, after adding 0.1 g of two-line ferrihydrite to solutions of DMMTA(V) and DMDTA(V) at pH of 4.0, 7.0, and 10.0. Their results showed that no species transformation occurred for DMMTA(V), and that DMDTA(V) was slightly oxidized to DMMTA(V) (approximately 4.2%) at pH 7.0. In the present study, as the Fe content of FeBC was approximately 12-fold lower than that of the two-line ferrihydrite used by Yoon et al. [12] (i.e., Fe content for 0.1 g of two-line ferrihydrite [5Fe_2_O_3_∙9H_2_O] was 58 mg [70], whilst Fe content for 0.1 g of FeBC was 4.72 mg), it was considered that no significant species transformation of DMMTA(V) and DMDTA(V) had occurred.

### 3.3. Chemical Forms of Adsorbed Dimethylated Arsenicals on Two-Line Ferrihydrite

**Figure 3:**
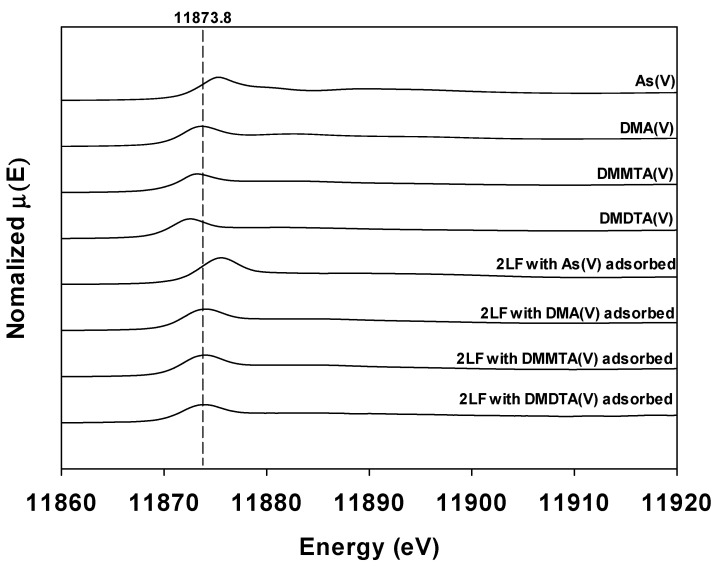
As reference compounds (As(V), DMA(V), DMMTA(V), and DMDTA(V)) and K-edge XANES spectra for two-line ferrihydrite, to which each As species was adsorbed. The dashed line represents the absorption energy of 11,873.8 eV.

An X-ray absorption near edge structure (XANES) analysis was performed to evaluate the chemical forms of dimethylated arsenicals adsorbed onto FeBC. As the main cause of the adsorption of dimethylated arsenicals onto FeBC was presumed to be the two-line ferrihydrite formed on the FeBC surface, the adsorption characteristics of dimethylated arsenicals on FeBC were studied by using the As K-edge XANES spectra of two-line ferrihydrite samples adsorbed with DMA(V), DMMTA(V), and DMDTA(V). Figure 3 shows the reference spectra of As(V), DMA(V), DMMTA(V), and DMDTA(V), as well as the K-edge XANES spectra of dimethylated arsenicals adsorbed on two-line ferrihydrite. The reference spectra of As(V), DMA(V), DMMTA(V), and DMDTA(V) showed absorption energies (white-line energy position) of 11,875.3, 11,873.8, 11,873.4, and 11,872.6 eV, respectively (Figure 3). It was confirmed that absorption energy decreased as the methyl and thiol group content increased. A similar decrease in absorption energy according to the increase of methyl and thiol group content has been reported previously (Table 3).

**Figure 4:**
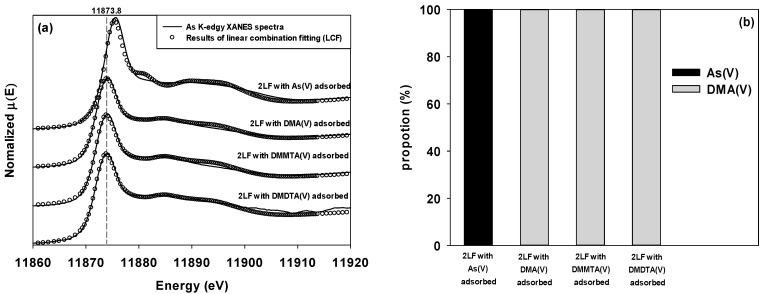
(**a**) LCF results of each As species adsorbed on two-line ferrihydrite (2LF) (open circles), against the As K-edge XANES spectra (solid lines). The R-factors for each analysis were 0.0105, 0.0014, 0.0027, and 0.0014 for the 2LF with adsorbed As(V), DMA(V), DMMTA(V), and DMDTA(V), respectively. (**b**) The stacked vertical bar indicates the ratio of As reference compounds (As(V), DMA(V), DMMTA(V), and DMDTA(V)) to compose the spectrum most similar to the spectrum of each sample, i.e., the proportion of As adsorbed to 2LF.

**Figure 5:**
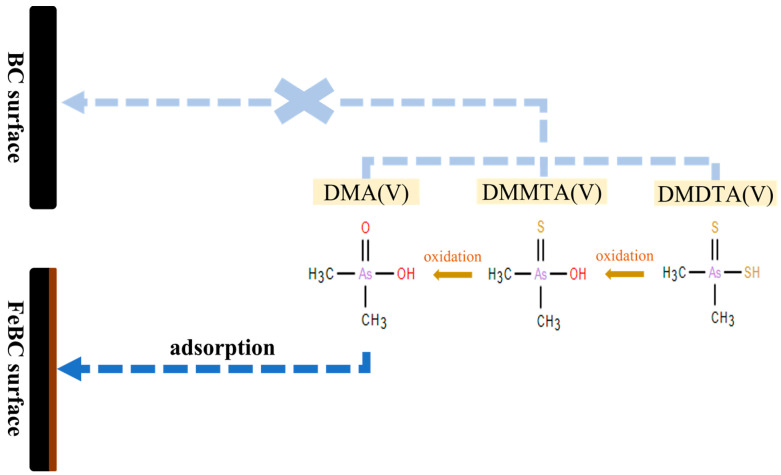
Proposed adsorption mechanisms of dimethylated arsenicals on FeBC.

In contrast, except for the two-line ferrihydrite adsorbed with As(V), the absorption energy of all samples adsorbed with DMA(V), DMMTA(V), and DMDTA(V) were the same as that of the reference compound (11,873.8 eV). Figure 4 shows the results of LCF for the As K-edge XANES spectra of each As species adsorbed on two-line ferrihydrite. The LCF results of the samples, in which each dimethylated arsenical was adsorbed on two-line ferrihydrite, indicated that the adsorbed As forms were all DMA(V) (Figure 4). Kim et al. [75] reported that ferric iron was able to serve as a catalyst to induce the oxidation of DMDTA(V) to DMMTA(V) and DMA(V), whilst Kerl et al. [76] noted that an estimated 50% of DMDTA(V) was converted to DMMTA(V) in an Fe-containing nutrient solution. Based on the findings of these studies, the results of the present study seemed to suggest that the partial oxidation of DMMTA(V) and DMDTA(V) may have occurred on the surface of FeBC, and that DMA(V) formed via oxidation was immediately adsorbed on two-line ferrihydrite formed on the surface of FeBC. As a result, the adsorbed dimethylated arsenicals appeared in the form of DMA(V) (Figure 5).

### 3.4. Environmental Implication

DMMTA(V) has been shown to exhibit high human- and eco-toxicity compared to DMA(V) and DMDTA(V). Cytotoxicity levels (IC50) of As(III), DMA(V), DMMTA(V), and DMDTA(V) in bladder cancer EJ-1 cells was reported to be 75 μM, ≥1 mM, 17 μM, and ≥1 mM, respectively [77], whilst the 48 h acute toxicities (IC50) of As(V), DMA(V), DMMTA(V), and DMDTA(V) against *Daphnia magna* was reported to be 9.5, <30, 1.7, and 6.5 mg/L, respectively [12]. In this study, it was confirmed that unmodified BC did not adsorb these highly toxic dimethylated arsenicals and that the adsorption capacity of BC for dimethylated arsenicals could be improved through surface modification with Fe oxides. Meanwhile, the coexistence of Fe oxides, such as two-line ferrihydrite and dimethylated arsenicals, induced transformation into DMA(V), which is less toxic in comparison to DMMTA(V) or DMDTA(V).

## 4. Conclusions

In this study, the adsorption characteristics of As(V) and dimethylated arsenicals (DMA(V), DMMTA(V), and DMDTA(V)) on BC and FeBC were assessed. As BC was modified with two-line ferrihydrite, the maximum adsorption capacity of As(V) increased approximately 5-fold, from 1.28 to 6.32 mg/g. This may have been due to the Fe oxide modification, thereby increasing adsorption sites on the FeBC surface and decreasing the electrostatic repulsion. Dimethylated arsenicals did not adsorb onto BC, whereas on FeBC, DMA(V), DMMTA(V), and DMDTA(V) showed maximum adsorption capacities at 7.08, 0.43, and 0.28 mg/g, respectively. As such, dimethylated arsenicals were adsorbed through the reaction with two-line ferrihydrite presented on the surface of FeBC. Similar to observations in previous studies, adsorption inhibition was observed with an increase in thiol-group content. The XANES-LCF analysis confirmed that the main form of dimethylated arsenicals adsorbed to two-line ferrihydrite was DMA(V). This indicated that FeBC could be utilized to lower the mobility of dimethylated arsenicals in the environment and to transform them into less hazardous forms.

## Figures and Tables

**Table 1:** Elemental mapping results (% surface Fe composition) for BC and FeBC, and total Fe concentration analyzed via aqua regia digestion.

Sample	Surface Fe Distribution (%) Determined Using EDS Elemental Mapping	Fe Concentration (mg/kg) Determined Using Aqua Regia Digestion and AAS Analysis
BC	0.2	527
FeBC	17.6	47,155

BC, biochar; FeBC, Fe/biochar composite; EDS, energy-dispersive X-ray spectrometer; AAS, atomic absorption spectrometer.

**Table 2:** Changes in adsorption capacity for anionic contaminants before and after Fe modification for various biomass-based adsorbents.

Biomass Used	Contaminant	Maximum Adsorption Capacity (mg/g)		Reference
		Biochar	Fe/Biochar Composite	
Hickory chips	Anionic dye (reactive red 120)	2.90	32.0	Feng et al. [33]
		2.39	79.4	
Corn stem	As(III)	2.89	8.25 (Fe-Mn modified biochar)	Lin et al. [35]
Pine wood	As(V)	0.265	0.429	Wang et al. [32]
Corn straw	As(V)	2.86	14.77	Fan et al. [34]
Activated carbon	As(V)	17.86	20.24	Yao et al. [36]
Empty fruit bunch	As(V)	5.5	15.2	Samsuri et al. [37]
	As(III)	18.9	31.4	
Rice husk	As(V)	19.3	30.7	
	As(III)	7.1	16.9	
Activated carbon	As(V)	0.00	4.663	Tuna et al. [38]
Rice straw	As(V)	0.552	5.923	Wu et al. [39]

BC, biochar; FeBC, Fe/biochar composite.

**Table 3:** Absorption energy (white-line energy position) (eV) of As species.

As Species	Absorption Energy (eV)	Reference
As(V)	11,875.3	Smith et al. [71]
As(V)	11,873.5	Jing et al. [72]
DMA(V)	11,873	
As(V)	11,875	Maher et al. [73]
DMA(V)	11,873	
Monothioarsenate	11,871.3	Suess et al. [74]
Dithioarsenate	11,870.3	
Tetrathioarsenate	11,869.8	
As(V)	11,873	Jeong et al. [69]
DMA(V)	11,872	
DMMTA(V)	11,871	
DMDTA(V)	11,870	

## Data Availability

The data are contained within the article.

## Supplementary Materials

The following supporting information can be downloaded at: https://www.mdpi.com/article/10.3390/toxics10110703/s1, Table S1: Parameters obtained through Langmuir model, Freundlich model, and Temkin model after isothermal adsorption experiments for each As species on FeBC.

## References

[1] Mohammed Abdul K.S., Jayasinghe S.S., Chandana E.P.S., Jayasumana C., De Silva P.M. (2015). Arsenic and human health effects: A Review. Environ. Toxicol. Pharmacol..

[2] Hughes M.F. (2002). Arsenic toxicity and potential mechanisms of action. Toxicol. Lett..

[3] Bondada B.R., Tu S., Ma L.Q. (2004). Absorption of foliar-applied arsenic by the arsenic hyperaccumulating fern (*Pteris vittata* L.). Sci. Total Environ..

[4] Vithanage M., Herath I., Joseph S., Bundschuh J., Bolan N., Ok Y.S., Kirkham M.B., Rinklebe J. (2017). Interaction of arsenic with biochar in soil and water: A critical review. Carbon.

[5] Parsons C.T., Couture R.-M., Omoregie E.O., Bardelli F., Greneche J.-M., Roman-Ross G., Charlet L. (2013). The impact of oscillating redox conditions: Arsenic immobilisation in contaminated calcareous floodplain soils. Environ. Pollut..

[6] Park S., Kim S.H., Chung H., An J., Nam K. (2022). Effect of organic substrate and FE oxides transformation on the mobility of arsenic by biotic reductive dissolution under repetitive redox conditions. Chemosphere.

[7] Garcia-Manyes S. (2002). Arsenic speciation in contaminated soils. Talanta.

[8] Han Y.-S., Park J.-H., Kim S.-J., Jeong H.Y., Ahn J.S. (2019). Redox transformation of soil minerals and arsenic in arsenic-contaminated soil under cycling redox conditions. J. Hazard. Mater..

[9] Thomas D.J. (2021). Arsenic methylation—Lessons from three decades of research. Toxicology.

[10] Katsoyiannis I., Zouboulis A. (2002). Removal of arsenic from contaminated water sources by sorption onto iron-oxide-coated polymeric materials. Water Res..

[11] Naranmandura H., Ibata K., Suzuki K.T. (2007). Toxicity of dimethylmonothioarsinic acid toward human epidermoid carcinoma A431 cells. Chem. Res. Toxicol..

[12] Yoon S.-G., Kim Y.-E., Chae C., An J., Yoon H.-O. (2021). Dimethylmonothioarsinic acid and dimethyldithioarsinic acid in the environment: Sorption characteristics on 2-line ferrihydrite and acute toxicity to daphnia magna. Environ. Geochem. Health.

[13] Planer-Friedrich B., London J., McCleskey R.B., Nordstrom D.K., Wallschläger D. (2007). Thioarsenates in geothermal waters of Yellowstone National Park:  Determination, preservation, and geochemical importance. Environ. Sci. Technol..

[14] Hug K., Maher W.A., Stott M.B., Krikowa F., Foster S., Moreau J.W. (2014). Microbial contributions to coupled arsenic and sulfur cycling in the acid-sulfide hot spring champagne pool, New Zealand. Front. Microbiol..

[15] Wang J., Kerl C.F., Hu P., Martin M., Mu T., Brüggenwirth L., Wu G., Said-Pullicino D., Romani M., Wu L. (2020). Thiolated arsenic species observed in rice paddy pore waters. Nat. Geosci..

[16] Li Y., Low G.K.-C., Scott J.A., Amal R. (2011). Microbial transformation of arsenic species in municipal landfill leachate. J. Hazard. Mater..

[17] Zaccone C., Lobianco D., Raber G., D’Orazio V., Shotyk W., Miano T.M., Francesconi K. (2018). Methylated arsenic species throughout a 4-m deep core from a free-floating peat island. Sci. Total Environ..

[18] Dai J., Chen C., Gao A.-X., Tang Z., Kopittke P.M., Zhao F.-J., Wang P. (2021). Dynamics of dimethylated monothioarsenate (DMMTA) in paddy soils and its accumulation in rice grains. Environ. Sci. Technol..

[19] Colina Blanco A.E., Kerl C.F., Planer-Friedrich B. (2021). Detection of thioarsenates in rice grains and rice products. J. Agric. Food Chem..

[20] Luvonga C., Rimmer C.A., Yu L.L., Lee S.B. (2020). Organoarsenicals in seafood: Occurrence, dietary exposure, toxicity, and risk assessment considerations—A Review. J. Agric. Food Chem..

[21] Zhang M., Gao B., Varnoosfaderani S., Hebard A., Yao Y., Inyang M. (2013). Preparation and characterization of a novel magnetic biochar for arsenic removal. Bioresour. Technol..

[22] Weber K., Quicker P. (2018). Properties of biochar. Fuel.

[23] Wang J., Wang S. (2019). Preparation, modification and environmental application of biochar: A Review. J. Clean. Product..

[24] Qian K., Kumar A., Zhang H., Bellmer D., Huhnke R. (2015). Recent advances in utilization of biochar. Renew. Sustain. Energy Rev..

[25] Tan X., Liu Y., Zeng G., Wang X., Hu X., Gu Y., Yang Z. (2015). Application of biochar for the removal of pollutants from aqueous solutions. Chemosphere.

[26] Alka S., Shahir S., Ibrahim N., Ndejiko M.J., Vo D.-V.N., Manan F.A. (2021). Arsenic removal technologies and future trends: A mini review. J. Clean. Prod..

[27] Montaño-Medina C.U., Lopéz-Martínez L.M., Ochoa-Terán A., López-Maldonado E.A., Salazar-Gastelum M.I., Trujillo-Navarrete B., Pérez-Sicairos S., Cornejo-Bravoc J.M. (2023). New pyridyl and aniline-functionalized carbamoylcarboxylic acids for removal of metal ions from water by coagulation-flocculation process. Chem. Eng. J..

[28] Zhou Z., Liu Y.-G., Liu S.-B., Liu H.-Y., Zeng G.-M., Tan X.-F., Yang C.-P., Ding Y., Yan Z.-L., Cai X.-X. (2017). Sorption performance and mechanisms of arsenic(v) removal by magnetic gelatin-modified biochar. Chem. Eng. J..

[29] Chen W.-H., Hoang A.T., Nižetić S., Pandey A., Cheng C.K., Luque R., Ong H.C., Thomas S., Nguyen X.P. (2022). Biomass-derived biochar: From production to application in removing heavy metal-contaminated water. Process Saf. Environ. Protect..

[30] Yaashikaa P.R., Senthil Kumar P., Varjani S.J., Saravanan A. (2019). Advances in production and application of biochar from lignocellulosic feedstocks for remediation of environmental pollutants. Bioresour. Technol..

[31] López-Maldonadoa E.A., Hernández-García H., Zamudio-Aguilar M.A.M., Oropeza-Guzmánc M.T., Ochoa-Teránc A., López-Martínezd L.M., Martinez-Quiroze M., Valdez R., Olivas A. (2020). Chemical issues of coffee and Tule lignins as ecofriendly materials for the effective removal of hazardous metal ions contained in metal finishing wastewater. Chem. Eng. J..

[32] Wang S., Gao B., Zimmerman A.R., Li Y., Ma L., Harris W.G., Migliaccio K.W. (2015). Removal of arsenic by magnetic biochar prepared from Pinewood and natural hematite. Bioresour. Technol..

[33] Feng K., Xu Z., Gao B., Xu X., Zhao L., Qiu H., Cao X. (2021). Mesoporous ball-milling iron-loaded biochar for enhanced sorption of Reactive Red: Performance and mechanisms. Environ. Pollut..

[34] Fan J., Xu X., Ni Q., Lin Q., Fang J., Chen Q., Shen X., Lou L. (2018). Enhanced As (v) removal from aqueous solution by biochar prepared from iron-impregnated Corn Straw. J. Chem..

[35] Lin L., Qiu W., Wang D., Huang Q., Song Z., Chau H.W. (2017). Arsenic removal in aqueous solution by a novel Fe-Mn modified biochar composite: Characterization and mechanism. Ecotoxicol. Environ. Saf..

[36] Yao S., Liu Z., Shi Z. (2014). Arsenic removal from aqueous solutions by adsorption onto iron oxide/activated carbon magnetic composite. J. Environ. Health Sci. Eng..

[37] Samsuri A.W., Sadegh-Zadeh F., Seh-Bardan B.J. (2013). Adsorption of As(iii) and As(v) by Fe coated biochars and biochars produced from empty fruit bunch and rice husk. J. Environ. Chem. Eng..

[38] Tuna A.Ö., Özdemir E., Şimşek E.B., Beker U. (2013). Removal of As(v) from aqueous solution by activated carbon-based hybrid adsorbents: Impact of experimental conditions. Chem. Eng. J..

[39] Wu C., Huang L., Xue S.-G., Huang Y.-Y., Hartley W., Cui M.-Q., Wong M.-H. (2017). Arsenic sorption by red mud-modified biochar produced from rice straw. Environ. Sci. Pollut. Res..

[40] Pedersen H.D., Postma D., Jakobsen R. (2006). Release of arsenic associated with the reduction and transformation of iron oxides. Geochim. Cosmochim. Acta.

[41] Jeong Y., Fan M., Singh S., Chuang C.-L., Saha B., Hans van Leeuwen J. (2007). Evaluation of iron oxide and aluminum oxide as potential arsenic(v) adsorbents. Chem. Eng. Process. Process Intensif..

[42] Mamindy-Pajany Y., Hurel C., Marmier N., Roméo M. (2009). Arsenic adsorption onto hematite and goethite. Comptes Rendus Chim..

[43] Song K., Kim W., Suh C.-Y., Shin D., Ko K.-S., Ha K. (2013). Magnetic iron oxide nanoparticles prepared by electrical wire explosion for arsenic removal. Powder Technol..

[44] Luther S., Borgfeld N., Kim J., Parsons J.G. (2012). Removal of arsenic from aqueous solution: A study of the effects of ph and interfering ions using iron oxide nanomaterials. Microchem. J..

[45] Jeong S., Yang K., Jho E.H., Nam K. (2017). Importance of chemical binding type between As and iron-oxide on bioaccessibility in soil: Test with synthesized two line ferrihydrite. J. Hazard. Mater..

[46] Su B., Lin J., Owens G., Chen Z. (2020). Impact of green synthesized iron oxide nanoparticles on the distribution and transformation of as species in contaminated soil. Environ. Poll..

[47] Tuutijärvi T., Lu J., Sillanpää M., Chen G. (2009). As(v) adsorption on maghemite nanoparticles. J. Hazard. Mater..

[48] Kim J., Song J., Lee S.-M., Jung J. (2019). Application of iron-modified biochar for arsenite removal and toxicity reduction. J. Ind. Eng. Chem..

[49] Li X., Qin Y., Jia Y., Li Y., Zhao Y., Pan Y., Sun J. (2021). Preparation and application of Fe/biochar (Fe-BC) catalysts in wastewater treatment: A review. Chemosphere.

[50] Liu H., Shan J., Chen Z., Lichtfouse E. (2021). Efficient recovery of phosphate from simulated urine by Mg/Fe bimetallic oxide modified biochar as a potential resource. Sci. Total Environ..

[51] Yi Y., Tu G., Zhao D., Tsang P.E., Fang Z. (2019). Biomass waste components significantly influence the removal of Cr(VI) using magnetic biochar derived from four types of feedstocks and steel pickling waste liquor. Chem. Eng. J..

[52] Reguyal F., Sarmah A.K., Gao W. (2017). Synthesis of magnetic biochar from pine sawdust via oxidative hydrolysis of FeCl2 for the removal sulfamethoxazole from aqueous solution. J. Hazard. Mater..

[53] Kang S., Kim G., Choe J.K., Choi Y. (2019). Effect of using powdered biochar and surfactant on desorption and biodegradability of phenanthrene sorbed to biochar. J. Hazard. Mater..

[54] Schwertmann U., Cornell R.M. (2001). Iron Oxides in the Laboratory Preparation and Characterization.

[55] An J., Jeong B., Nam K. (2019). Evaluation of the effectiveness of in situ stabilization in the field aged arsenic-contaminated soil: Chemical extractability and biological response. J. Hazard. Mater..

[56] Park J., Chung H., Kim S.H., An J., Nam K. (2020). Effect of neutralizing agents on the type of as co-precipitates formed by in situ Fe oxides synthesis and its impact on the bioaccessibility of as in Soil. Sci. Total. Environ..

[57] Xu X., Zheng Y., Gao B., Cao X. (2019). N-doped biochar synthesized by a facile ball-milling method for enhanced sorption of CO_2_ and reactive red. Chem. Eng. J..

[58] Yao Y., Gao B., Zhang M., Inyang M., Zimmerman A.R. (2012). Effect of biochar amendment on sorption and leaching of nitrate, ammonium, and phosphate in a sandy soil. Chemosphere.

[59] Mukherjee A., Zimmerman A.R., Harris W. (2011). Surface Chemistry variations among a series of laboratory-produced biochars. Geoderma.

[60] He R., Peng Z., Lyu H., Huang H., Nan Q., Tang J. (2018). Synthesis and characterization of an iron-impregnated biochar for aqueous arsenic removal. Sci. Total. Environ..

[61] Pham T.H., Chu T.T.H., Nguyen D.K., Le T.K.O., Obaid S.A., Alharbi S.A., Kim J., Nguyen M.V. (2022). Alginate-modified biochar derived from rice husk waste for improvement uptake performance of lead in wastewater. Chemosphere.

[62] Ahmad A., Khan N., Giri B.S., Chowdhary P., Chaturvedi P. (2020). Removal of methylene blue dye using rice husk, cow dung and sludge biochar: Characterization, application, and kinetic studies. Bioresour. Technol..

[63] Cismasu A.C., Michel F.M., Tcaciuc A.P., Tyliszczak T., Brown G.E. (2011). Composition and structural aspects of naturally occurring ferrihydrite. C. R. Geosci..

[64] López-Maldonadoa E.A., Oropeza-Guzmán M.T. (2021). Nejayote biopolyelectrolytes multifunctionality (glucurono ferulauted arabinoxylans) in the separation of hazardous metal ions from industrial wastewater. Chem. Eng. J..

[65] Kuppler R.J., Timmons D.J., Fang Q.-R., Li J.-R., Makal T.A., Young M.D., Yuan D., Zhao D., Zhuang W., Zhou H.-C. (2009). Potential applications of metal-organic frameworks. Coord. Chem. Rev..

[66] Rivadeneira-Mendoza B.F., Filho O.A.E., Fernández-Andrade K.J., Curbelo F., Silva F.F.D., Luque R., Rodríguez-Díazhi J.M. (2023). MOF@biomass hybrids: Trends on advanced functional materials for adsorption. Environ. Res..

[67] Dai Y., Zhang N., Xing C., Cui Q., Sun Q. (2019). The adsorption, regeneration and engineering applications of biochar for removal organic pollutants: A review. Chemosphere.

[68] Rosales E., Meijide J., Pazos M., Sanromán M.A. (2017). Challenges and recent advances in biochar as low-cost biosorbent: From batch assays to continuous-flow systems. Bioresour. Technol..

[69] Jeong S., Kang J., Cho M., An J., Yoon H.-O. (2022). New insights into surface behavior of dimethylated arsenicals on montmorillonite using X-ray absorption spectroscopy. Sci. Total Environ..

[70] Das S., Hendry M.J., Essilfie-Dughan J. (2010). Transformation of two-line ferrihydrite to goethite and hematite as a function of pH and temperature. Environ. Sci. Technol..

[71] Smith P.G., Koch I., Gordon R.A., Mandoli D.F., Chapman B.D., Reimer K.J. (2004). X-ray absorption near-edge structure analysis of arsenic species for application to biological environmental samples. Environ. Sci. Technol..

[72] Jing C., Meng X., Liu S., Baidas S., Patraju R., Christodoulatos C., Korfiatis G.P. (2005). Surface complexation of organic arsenic on nanocrystalline titanium oxide. J. Colloid Interface Sci..

[73] Maher W., Foster S., Krikowa F., Donner E., Lombi E. (2013). Measurement of inorganic arsenic species in rice after nitric acid extraction by HPLC-ICPMS: Verification using Xanes. Environ. Sci. Technol..

[74] Suess E., Scheinost A.C., Bostick B.C., Merkel B.J., Wallschlaeger D., Planer-Friedrich B. (2009). Discrimination of thioarsenites and thioarsenates by X-ray absorption spectroscopy. Anal. Chem..

[75] Kim Y.-T., Lee H., Yoon H.-O., Woo N.C. (2016). Kinetics of dimethylated thioarsenicals and the formation of highly toxic dimethylmonothioarsinic acid in environment. Environ. Sci. Technol..

[76] Kerl C.F., Schindele R.A., Brüggenwirth L., Colina Blanco A.E., Rafferty C., Clemens S., Planer-Friedrich B. (2019). Methylated thioarsenates and monothioarsenate differ in uptake, transformation, and contribution to total arsenic translocation in rice plants. Environ. Sci. Technol..

[77] Naranmandura H., Carew M.W., Xu S., Lee J., Leslie E.M., Weinfeld M., Le X.C. (2011). Comparative toxicity of arsenic metabolites in human bladder cancer EJ-1 cells. Chem. Res. Toxicol..

