# Supplementary material for: Adsorption Characteristics of Dimethylated Arsenicals on Iron Oxide–Modified Rice Husk Biochar
Source: Toxics. 2022 Nov 17;10(11):703. doi: 10.3390/toxics10110703 (PMC9692524; doi:10.3390/toxics10110703)
Supplement: Supplementary file 1 [file toxics-10-00703-s001.zip › toxics-1969921-supplementary.pdf]

Supplementary Materials:
Adsorption characteristics of dimethylated arsenicals on iron oxide-modified rice husk biochar

Sang-Gyu Yoon[1], Ihn-Sil Kwak[2], Hye-On Yoon[3*], Jinsung An[4*]

Table S1. Parameters obtained through Langmuir model, Freundlich model, and Temkin model after isothermal adsorption experiments for each As species on FeBC

Model	Equation	Parameter	As(V)	DMA(V)	DMMTA(V)	DMDTA(V)
Langmuir	$q_e = \frac{q_m b C_e}{1 + bC_e}$	q_m (mg/g)	6.32	7.08	0.430	0.280
		b (L/mg)	0.023	0.002	0.013	0.115
		R^2	0.684	0.792	0.508	0.772
Freundlich	$q_e = K_F C_e^{1/n_f}$	K_F ($mg^{(1-1/n)}$ $L^{1/n}$/g)	1.83	0.286	0.009	0.050
		n_f	5.65	2.65	1.38	2.42
		R^2	0.682	0.671	0.466	0.688
Temkin	$q_e = \frac{RT}{B} \ln A_T C_e$	B (kJ/mol)	2.49	1.95	47.4	45.6
		A_T (L/mol)	0.597	0.031	0.439	1.51
		R^2	0.789	0.725	0.391	0.748

K_F: the Freundlich constant

n_f: the heterogeneity factor

B: the constant related to the adsorption heat

A_T: the equilibrium binding constant
